# Manual of Human Microscopical Anatomy

**Published:** 1855-04

**Authors:** 


### REVIEW DEPARTMENT.

### ARTICLE XIV.

*Manual of Human Microscopical Anatomy.* By A. KÖLLIKER, Professor of Anatomy and Physiology in Würtzburg. Translated by GEORGE BUSK, F. R. S. and THOMAS HUXLEY, F. R. S. Edited with notes and additions, by J. DA COSTA. M. D. Philadelphia, Lippincott, Grambo & Co., 1854.

THE microscope has not only widened the sphere of our observation, and increased the objective basis of our knowledge, but it has also, in these late years, aided us in our efforts to penetrate the secrets of organization and to study the philosophy of life. Originally a scientific toy, fit only to rouse the feeble raptures of St. Pierre or the shallow enthusiasm of Rousseau, it has become the means of opening to the scientific eye, depths of existence previously unknown, and raising the mind to heights of speculation hitherto unsuspected.

The assiduity with which it is now used, the earnestness with which its revelations are studied, the closeness with which nature has been cross-questioned by its votaries, have produced their necessary results. An enormous mass of new facts has been disentombed. Some of them have been classified; others are now awaiting classification. A great portion still remains "rudis indigestaque moles," but already on scattered points of the vast abyss an orderly accretion is seen to be going on.

This is not the place to speak of the geological applications of this wondrous instrument, to show how it has wrenched from the dead Past the most guarded secrets of its stupendous cycles. The dust of Bilin, and the sand of Richmond would to this day have remained dust and sand without its aid. Chemistry indeed suspected the origin of coal, but it was left for the microscope to unfold the minute structure of these great deposits, to demonstrate, beyond all possibility of doubt, their vegetable origin, and to enable the eye of imagination to look back upon that dense forest of fantastic forms which shaded the dank mould of the primeval earth.

Far more interesting to us are those revelations of existing structure which the microscope affords. Histology lives only in the tube of this optical instrument. It is a science of the last two centuries, and is yet far from its maturity. Its greatness is all in the future. Comprising, as it does, not only the history of existing forms of ultimate organization, but the origin, development, growth, maturity and decay of those minute parts of which it takes cognizance, it is manifestly no ordinary labor which devolves upon its votaries.

It is important, here, as every where else in science, to survey the ground which lies before us and thoroughly to understand the capabilities of our instruments and the extent of their revelations. It is of the last importance to become acquainted with the narrowness of that sphere in which we are working, for what, after all, is the result of our laborious study but to discover the limits of our ignorance?

So far as the scope and progress of histology are concerned, the author we are reviewing has stated it so clearly and in so condensed a form that we can do no better than quote his observations.

"In characterizing the present position of histology and of its objects, we must by no means forget that, properly speaking, it considers only one of these aspects which elementary parts present to observation, namely, their form. Microscopical anatomy is concerned with the understanding of the microscopic forms, and with the laws of their structure and development, not with any general doctrine of the elementary parts. Composition and function are only involved, so far as they relate to the origin of forms and their variety, whatever else respecting the activity of the perfect elements and their chemical relations is to be found in histology, is there either on practical grounds in order to give some useful application of the morphological conditions, or to complete them; or from its intimate alliance with the subject, it is added only because physiology proper does not afford a due place for the functions of the elementary parts.

"If histology is to attain the rank of a science, its first need is to have as broad and certain an objective basis as possible. To this end the minuter structural characters of animal organisms are to be examined on all sides, and not only in fully formed structures, but in all the earlier periods from their first development when the morphological elements have been perfectly made out; the next object is to discover the laws according to which they arise, wherein we must not fail to have regard also to their relations of composition and functions. In discovering these laws, here as in the experimental sciences generally, continual observation separates more and more, among the collective mass of scattered facts and observations, the occasional from the constant, the accidental from the essential, till at last a series of more and more general expressions of the facts arises, from which, in the end, mathematical expressions or formulæ proceed, and thus the laws are enunciated.

"If we inquire how far histology has satisfied these requirements, and what are its prospects in the immediate future, the answer must be a very modest one. *Not only does it not possess a single law*, but the materials at hand from which such should be deduced, are as yet relatively so scanty, that not even any considerable number of general propositions appear well founded. Not to speak of a complete knowledge of the minuter structure of animals in general, we are not acquainted with the structure of a single creature throughout, not even of man, though he has so frequently been the object of investigation—and, therefore, it has hitherto been impossible to bring the science essentially any nearer its goal. It would, however, be unjust to overlook and depreciate what we do possess; and it may at any rate be said that we have acquired a rich store of facts and a few more trustworthy general propositions. To indicate only the more important of the former, it may be mentioned that we have a very sufficient acquaintance with the perfect elementary parts of the higher animals, and that we also understand their development, with the exception of the elastic tissue, and of the elements of the teeth and bones. The mode in which these are united into organs has been less examined, yet on this head also, much has been added of late, especially in man, whose individual organs, with the exception of the nervous system, the higher organs of sense, and a few glands (the liver, blood-vascular glands) have been almost exhaustively investigated. If the like progress continue to be made, the structure of the human body will, in a few years, be so clearly made out, that except perhaps in the nervous system, nothing more of importance will remain to be done with our present modes of investigation. With comparative histology it is otherwise; hardly commenced, not years but decades will be needed to carry out the necessary investigations. Whoever will do good in this field must, by monographs of typical forms, embracing their whole structure from the earliest period of development, obtain a general view of all the divisions of the animal kingdom, and then by the methods above described, strive to develop their laws."

We have given space to this long quotation, because we think it covers very important ground, and that it indicates the true method of advancing this interesting department of science. As to general histology, Kölliker thinks it has made no important progress since Schwann.

In regard to the instruments to be used, the author recommends those made by Plössel, Oberhäuser and Schiek. He considers Nachet's microscopes good, but inferior to those of Oberhäuser, and admits those of Ross, Powell and Lealand, to be quite equal "to Oberhäuser's," "but out of the question for Germany," on account of their high price, we presume. The American editor, Dr. Da Costa, of Philadelphia, regards his author's opinion as "entirely too national," and considers the English instruments as "far superior" to the German. Nachet's are economical, and are undoubtedly the most used in this country and in England. We wish, however, that the American editor had been a little more national, in his estimate of Spencer's instruments, the lenses of which he characterises as "not inferior to the best glasses either of Ross or of Powell and Lealand." We had certainly expected from an American editor some account of the remarkable enlargement of the angle of aperture and the perfect illumination of the field in these American instruments, and think that our eminent optician has been passed over far too slightly by Dr. Da Costa.. The sensation which these remarkable objectives have created not only in scientific circles in our own country, but among the most distinguished men abroad, leaves the American microscopist without excuse, who can fail to state their eminent superiority. We have already said so much about these instruments of Spencer in this Journal, that it can hardly be necessary for us to add any thing in this place, and we content ourselves with referring those who desire particular information on this subject, to Dr. Dwinelle's articles on the microscope in previous numbers, especially to that published last January, in which the value of these objectives is very fully discussed.

The plan of the work does not materially differ from that upon which histological disquisitions are commonly constructed. After the introduction, from which we have quoted, containing an account of the history and present position of the science, with a discussion of the aids to the study, follows the division of general histology. This is divided into two portions, the first, treating of the elementary parts, the second of the tissues, organs and systems. The first are divided into simple and compound or higher elementary parts.

Under the head of simple parts, are described the elementary granules, the vesicles and nuclei, as well as the cells. To these great space is given, and we have a very satisfactory account of the form, chemical character and vital functions of cells. The recent researches of Donders are embodied in this chapter. We have not the space to enter into a consideration of them, and can only refer our readers to the work itself. We cannot, however, refrain from alluding to the interpretation put upon the elasticity of the cell-membranes. This property of course gives a varying pressure upon the cell-contents, in accordance with their variable states of fullness. The greater the pressure the greater the excretion, and on the other hand, the less the pressure, the greater the absorption.

The higher elementary parts are made of a series of simple ones, the cells only according to this authority, being capable of producing them. These higher parts are the cell-fibres and cell-networks, the elastic fibres and fibrous networks, the fibres, networks and membranes of connective tissue, the striated muscular fibres, the nerve fibres and their networks, the capillary plexuses of the blood-vessels and lymphatics, and the tracheæ of insects.

The tissues are also classified under the two heads of simple and compound. Under the first head, we find four tissues; the epidermic; the cartilaginous; the elastic and yellow fibrous; and the connective or white fibrous tissue. The complex tissues are the osseous; the smooth muscular; the transversely striated muscular; the nervous; the tissue of the blood-vascular glands, and that of the true glands. In all, therefore, according to this classification, there are ten tissues, six of which are compound and four simple.

After this follows special histology, under which the various systems are described in the usual order.

Our space is too limited to take even a cursory view of the numerous subjects treated in this work; we shall therefore be compelled to confine ourselves to those portions of the book likely to prove most interesting to our readers, and these, we regret to say, we shall, for the same reasons, be compelled to pass over far more rapidly than we desire.

No less than 78 pages are accorded to bone and 32 to the teeth in the present edition of this work. In both these chapters the English translators have made large additions to the text, in the form of notes, embodying the recent researches of English microscopists.

In the annotations to the chapter on osseous tissue, frequent reference is made to the recent paper of Tomes and De Morgan on this subject. These writers have described, in addition to the universally recognized Haversian canals, other cavities in bone, which they denominate ***Haversian spaces.*** These are easily distinguished from the canals by their outline, which, instead of being smooth and rounded, like the latter, is irregular, like that of the surface of exfoliations. Besides, the laminæ of the canal are arranged in a more or less conformable manner around it, while the walls of the spaces are formed by the unconformable edges of the laminæ of the adjacent Haversian canals, which have been eaten away to form the space. This is the product of absorption, and the formation of the cavity is, in its turn, arrested, when new depositions of bony matter take place within it, and are traversed by new canals that ultimately give way again before the process of absorption, when these new canals have been narrowed to a certain point. Bone, therefore, is no stationary tissue, but one undergoing active molecular change, as we might infer from its high vascularity, and the history of its processes of disease and repair.

The structure of the laminæ is best seen in moistened sections. When soaked with oil of turpentine, each laminæ is seen to be composed of two layers, an outer, highly granular, often composed of a single series of large granules, and an inner, clear, transparent, and apparently structureless. This division, however, does not always exist, and often in the innermost lamina of a Haversian canal the whole layer is clear, glassy, and without apparent structure.

The striated appearance seen in the laminæ, and formerly believed to indicate a fibrous structure, is considered by Tomes and De Morgan to arise from imperfect illumination and definition. The alternate structure is believed by these authors to consist of minute granules or granular cells imbedded in a more or less clear, homogeneous, or subgranular matrix.

The circumferential laminæ, according to the same observers, are not so constantly present as is generally supposed, and rarely entirely surround the shaft of a long bone. They are found only in adults, and then not always distinctly made out, so that their presence seems to indicate an arrest of growth, though not, of course, of interstitial change. In young, rapidly growing bone, on the contrary, these circumferential laminæ are replaced by a series of *undulating laminæ,* so that the surface is formed of a series of re-duplicated processes, arching over and enclosing the nearest vessels of the periosteum.

In the description of the lacunæ we observe nothing new, except the account of their contents. These appear to be a nucleus and a clear viscid fluid like that contained in cartilage cells. The nuclei may be brought out by treatment with acetic or strong hydrochloric acid. Tomes and De Morgan state that they have been able to see them even in fossil bone.

As to the *teeth*, we find a summary of the various discordant opinions of different observers in a note of the translators on the subject of dentine. We quote only the observations of Mr. James A. Salter, who considers the contour-lines to arise not only from curvings and local enlargements of the canals, and interlobular spaces, but also from a difference in density, and he states that these curves cross the primary curves of the dentinal tubes at right angles. No markings are more divergent than the outline of the tooth, and passing from within outwards, they abut in succession upon the external surface of the dentine, under the enamel and crusta petrosa, in the form of granular patches. The outer extremities of these patches look like white rings on the surface of the teeth. They are composed of coarse globular dentine, and gradually thin out internally into mere streaks. When a tooth is macerated in acid, it may be broken up into cones formed by the normal dentine between the contour markings. In transverse sections, the cones become, of course, rings. Finally, the enamel is almost always imperfect, opposite the "patches" at the outer end of the contour line.

The following account of dental caries is interesting. "It attacks living and false teeth, and always begins in the exterior, from Nasmyth's membrane, whence the fluids of the mouth have been supposed to have considerable influence upon it; it does not follow, however, that one living tooth may not be more disposed to it than another, being rendered less capable of resistance, either by its chemical composition, or by the mode of its nutrition. Caries, however, is assuredly not a simple solution of the salts by the oral fluids, but a solution accompanied by a putrefactive decomposition of the organic elements of the tooth, which becomes covered with infusoria and fungi; in fact, according to Ficinus' observations, the latter growth would appear to play the more important part, inasmuch as the decay of the teeth usually commences in those localities in which undisturbed opportunity is given to those organisms to develop, as in the cracks and pits of the enamel, in the depressions of the molar teeth, in the clefts between the teeth, but not where the dentine is otherwise exposed, as on the masticating surface, in filed places, &c. The usual cause of caries is, that the discolored spots of the cuticle of the enamel, covered with living and growing organisms, (infusorial animalcules, similar to a vibrio, which Ficinus calls *denticola*, mucedinous fungi, similar to those which are found upon the tongue, and which Ficinus wrongly refers to the *denticolæ*,) first lose their calcareous salts, and then break up into angular, cellular pieces, as if they had been treated with hydrochloric acid. The decay then penetrates through the enamel to the dentine, always first softening it, so that it yields not more than ten per cent. of ashes, then decomposing it. The dentine is more affected by this process than the enamel, its canal first becoming filled with the fluids proceeding from its decomposition, which may reach the pulp, and give rise to pain, unless, as Tomes found, the dentinal canals in the neighboring healthy portions become obliterated by deposits, or the pulp is protected by new masses of dentine developed in the cavity. Eventually a brownish deposit takes place in the tubules, and then the intermediate substance becomes completely broken up. In this manner the process of decomposition extends further and further, until at last the crown collapses, the root also becoming dissolved, and finally falling out."

We are now compelled unwillingly to take leave of this book, having but given a hurried glance to its contents. We cannot close these hasty remarks, however, without commending the manner in which the mechanical execution of the book has been attended to. It is printed on fine paper, in a clear type, and the numerous wood cuts are generally well done and properly printed—a compliment we cannot conscientiously pay to many of our illustrated books. We ought also to mention that the bibliography appended to the different sections, though not very full, is carefully selected, and will be found an important aid to the student who desires to prosecute more extended researches.

